# Cooperation without punishment

**DOI:** 10.1038/s41598-023-28372-y

**Published:** 2023-01-21

**Authors:** Balaraju Battu, Talal Rahwan

**Affiliations:** Science Division, New York University Abu Dhabi, Abu Dhabi, UAE

**Keywords:** Computational biology and bioinformatics, Evolution, Environmental social sciences

## Abstract

A fundamental question in social and biological sciences is whether self-governance is possible when individual and collective interests are in conflict. Free riding poses a major challenge to self-governance, and a prominent solution to this challenge has been altruistic punishment. However, this solution is ineffective when counter-punishments are possible and when social interactions are noisy. We set out to address these shortcomings, motivated by the fact that most people behave like conditional cooperators—individuals willing to cooperate if a critical number of others do so. In our evolutionary model, the population contains heterogeneous conditional cooperators whose decisions depend on past cooperation levels. The population plays a repeated public goods game in a moderately noisy environment where individuals can occasionally commit mistakes in their cooperative decisions and in their imitation of the role models’ strategies. We show that, under moderate levels of noise, injecting a few altruists into the population triggers positive reciprocity among conditional cooperators, thereby providing a novel mechanism to establish stable cooperation. More broadly, our findings indicate that self-governance is possible while avoiding the detrimental effects of punishment, and suggest that society should focus on creating a critical amount of trust to harness the conditional nature of its members.

## Introduction

Is self-governance possible when collective and private interests are at odds? This has been a fundamental question in social and biological sciences^1–6^. Public good provision provides a context in which such conflict can be studied^7^. Optimal public good provision requires all individuals to contribute maximally towards the common pool. However, the non-excludable nature of the common good incentivizes selfish individuals to free ride on the contribution of others, leading to an inefficient outcome. For decades, the challenge posed by free-riders has been the subject of intense investigation^3,4,8–21^. To address this challenge, voluntary cooperation has been studied experimentally^22^ and theoretically^23^ using the public goods game (PGG). In a typical PGG, each individual is given an equal amount of endowment, along with the option to contribute voluntarily and anonymously to a common pool. After the contribution decisions are made, the collected endowment is enhanced and is divided equally among all individuals, irrespective of their contributions to the common pool. Essentially, a PGG is an n-person prisoners’ dilemma, and according to standard game theory, individuals choose their strategy under the assumption of rationality and common knowledge^24^. This implies that individuals maximize their payoff by not contributing to the common pool, leading to the *tragedy of the commons*^25^. However, contrary to the predictions made by standard game theory, behavioural experiments on the PGG have shown that most people contribute to the common pool, and only a few individuals free ride^16,16,26,27^. Yet, when the PGG is repeated over multiple rounds, contributions tend to decline with the number of rounds; this is due to a few individuals who free ride in the first round, thereby triggering further free riding in subsequent rounds^11,14,27^. Most proposed solutions to this problem involve either pool punishment^28,29^ or peer punishment^10,11,15,15,18,19,30–33^. A prominent form of the latter is altruistic punishment, whereby individuals punish free-riders at a personal cost. It has been observed that people are often willing to engage in altruistic punishment, even when the cost to the punisher is substantial^14,15^.

Unfortunately, numerous studies have shown that punishment-based mechanisms only solve the free-rider problem under restrictive conditions^34–38^. Specifically, pool punishment is ineffective when the cost of maintaining central authority outweighs the benefits of cooperation. Moreover, in scenarios where there are no free riders, community members may lack the incentive to contribute towards maintaining the central authority. Another limitation of pool punishment is information loss. In particular, when information is transmitted from the local commons all the way up the hierarchy, some information may be misinterpreted, potentially leading the central authority to undermine the sentiments of the local community^9,21,39^. As for peer punishment, it is ineffective when the population is dominated by second-order free-riders, i.e., individuals who contribute to the public goods but do not punish free-riders^40,41^. As far as altruistic punishment is concerned, it is ineffective when the possibility arises for punishing the cooperators, e.g., due to bribery^42^ or imperfect information^43^. Moreover, this mechanism does not distinguish between those who free ride due to selfish interests and those who free ride only because others are doing so^44^. Overall, punishment can override altruistic motives, leading individuals to free ride as soon as the opportunity arises to evade punishment^45–47^.

The literature on cooperation explores various mechanisms that do not rely on punishment. These include cognitive factors such as intuition and deliberation^48–50^, as well as psychological factors, such as imitation of emotions^51,52^, positive interactions^53,54^, and rewards^55–58^. Other mechanisms include the coevolution of norms^59,60^, the coevolution of costs and benefits^61^, and opting out from the public good game altogether^62–64^. However, none of these mechanisms exploit positive reciprocity and the conditional nature of human cooperation. Conditional cooperators react to the actions of others in the population. For example, in collective risk dilemmas and public goods games, individuals’ non-binding pledges in the population can trigger conditional cooperation in subsequent rounds^17,65^. However, even if social groups manage to successfully trigger conditional cooperation, they would still have to overcome the challenge of sustaining that cooperation. This challenge stems from the fact that a few selfish free-riders can always trigger negative reciprocity from the conditional cooperators, thereby spreading the free riding behaviour across the population. This proliferation can be explained in terms of evolutionary biology, where payoff is equated with fitness, and relatively higher fitness agents—the free-riders—reproduce faster than others^66,67^. It can also be explained in terms of cultural evolution, whereby individuals imitate the social behaviour of successful role models. Thus, to sustain cooperation, we need a mechanism to slow down the proliferation of free-riders.

Our novel alternative to punishment builds on the reciprocal nature of the trust—the fact that people reciprocate voluntary trust^68^—as well as the conditional nature of human cooperation—the fact that negative reciprocity can be switched to positive reciprocity given sufficient cooperation levels^44,69–71^. More specifically, it has been observed that most people reciprocate trust when the initial act of trust is taken voluntarily^68^, e.g., as is the case with successful business organizations who foster a culture of trust by placing trust in others^72–74^. Moreover, it has also been observed that, in repeated public goods games, most people behave like conditional cooperators—individuals willing to cooperate if a critical number of others do so—and only a few individuals tend to be altruists (i.e., unconditional cooperators) or free-riders^16,17,27,75,76^. Based on this observation, instead of penalizing the selfish tendencies of the minority—as is the case with punishment—our proposed mechanism involves placing a few altruists in the population, thereby harnessing the conditional nature of the majority. We hypothesize that, in the presence of a few altruists, initial trust can be created, thereby triggering positive reciprocity among conditional cooperators. The idea of influencing a population, not via punishment but rather via the actions of a few individuals, has been discussed in the literature, albeit not in the context of cooperation. More specifically, it has been shown that a population’s opinion can be influenced by a few “stubborn individuals”^77^, and social norms can be changed by a few deviants or cultural leaders^78–80^.

Using an evolutionary agent-based model, we show that, in the presence of altruists and moderately noisy social interactions, it is possible for a population of conditional cooperators to establish and sustain high levels of cooperation. The altruists trigger positive reciprocity, and the combination of altruists and moderate levels of noise provides an environment under which social learning is not only influenced by *payoff-biased transmission*—the tendency to copy the most successful individual—but also influenced by *conformist-biased transmission*—the tendency to imitate the most frequent behaviour in the population—thereby establishing and sustaining cooperation. Importantly, unlike punishment, our mechanism can sustain cooperation under a wide range of noise in social interactions, whereby agents can occasionally commit mistakes in their conditional cooperative decisions and their adaptation of the social behaviour of role models. More broadly, we show that stable cooperation can be established, not by punishment or reward, but rather by creating initial trust in the population, thereby exploiting the conditional nature of human cooperation.

## Model

Our evolutionary agent-based model involves multiple generations. In each generation, the agents play a PGG, after which they update their strategies. Next, we explain this model in more detail.

### Population type

The population consists of *N* agents, with a small fraction consisting of *altruists* (i.e., agents who cooperate unconditionally), and the rest consisting of *conditional cooperators* (i.e., agents who are willing to cooperate if others do so); this resembles behavioural regularities observed elsewhere^17,81^. More specifically, each agent, *i*, is born into the population with a conditional cooperative criterion, $$ CCC _i \in \{0,\ldots ,N\}$$, which specifies the agent’s conditional rule, i.e., the minimum number of cooperation decisions required for *i* to cooperate. For example, if $$CCC_i=m$$, then *i* may cooperate in the current round if and only if the number of agents who cooperated in the previous round is $$\ge m$$. Now, for any altruist *j*, we have $$ CCC _j = 0$$. On the other hand, for any conditional cooperator *k*, we have $$ CCC _k \sim \{0,\ldots ,N\}$$. Clearly, if $$ CCC _k=0$$ then *k* always cooperates, and if $$ CCC _k = N$$ then *k* always free rides. Crucially, however, even if $$ CCC _k = 0$$, it does not make *k* an altruists. More specifically, given an altruist *j* and a conditional cooperator *k* such that $$ CCC _j = CCC _k = 0$$, although the two agents would cooperate unconditionally in the current generation, they would still differ in the way they update their strategies after each generation, as we will see in the coming section on updating the population.

### Conditional cooperative decisions

In any given round, the cooperative decision of agent *i* depends on $$ CCC _i$$ as well as the number of agents who contributed in the previous round, denoted by $$N_C$$. More specifically, in the current round, *i* contributes to the common pool with a probability:

1 $$\begin{aligned} p_i = \left\{ \begin{array}{ll} 1 &{} \text {if } CCC_i = 0 \\ \frac{1}{1+\exp \left( -\left( N_C - CCC_i\right) \beta \right) } &{} \text {otherwise} \end{array} \right. \end{aligned}$$

where $$\beta \ge 0$$ represents the noise level, i.e., the uncertainties in making a conditional decision. As $$\beta $$ approaches infinity, the noise level approaches 0, in which case agent *i* has perfect information about $$N_C$$, and its decision depends solely on its conditional rule, i.e., it contributes if and only if $$(N_C - CCC _i) > 0$$. When either $$\beta = 0$$ or $$(N_C- CCC _i)=0$$, the agent decides whether to contribute by tossing a coin. The rationale behind this design decision is as follows. When the former condition is met, i.e., when $$\beta = 0$$, all information is lost, implying that the agent is unable to access any information about $$N_C$$. In contrast, when the latter condition is met, i.e., when $$(N_C - CCC _i) = 0$$, the agent is confused about whether or not to cooperate. For intermediate values, i.e., when $$0< (N_C - CCC _i)\beta < 1$$, the agent contributes to the public good with probability $$0.5< p_i < (e / (e + 1))$$. We can infer that, given the same level of cooperation in the previous round, agents with larger $$ CCC _i$$ values are less likely to contribute than those with smaller values. Placing a certain percentage of altruists in the population would increase $$N_C$$, thereby triggering positive reciprocity from the conditional cooperators. The combination of CCC values of the agents and $$\beta $$ mimics the behavioural regularities observed in repeated public goods games elsewhere^17,81^.

### The public goods game

All the agents play a public goods game in each generation. At the beginning of the PGG, each agent is given an equal endowment, *e*. Then, following Eq. (1), agent *i*’s contribution to the public good is $c_i\in \{0,u\}$ where $0<u\le e$; we refer to *u* as the contribution cost. After all the individuals make their contributing decisions, the payoff of agent *i* would be:

2 $$\begin{aligned} \pi _i = \left( e - c_i \right) + \frac{h}{N}\sum \limits _{i=1}^{N} c_i \end{aligned}$$

where $h > 1$ is the enhancing factor of the collective good. From this equation, one can see that the free riding yields higher payoffs than contributing to the public good.

### Updating the population

Every conditional cooperator *i* updates its $$CCC_i$$ value after each generation, i.e., after each round of the PGG. The update is done using a pair-wise comparison process^82^. First, *i* is matched with another randomly-selected agent *j*. Then, if *j* happens to be a role model of *i* (i.e., if $$\pi _j > \pi _i$$ in the previous generation), then *i* adapts *j*’s strategy (i.e., it sets $$ CCC _i = CCC _j$$) with a probability $$q_i$$ which is proportional to the payoff difference between *i* and *j*. More formally:

3 $$\begin{aligned} q_i = \frac{1}{1+\exp \left( -\left( \pi _j - \pi _i\right) \beta \right) } \end{aligned}$$

In this process, high-fit agents can reproduce two offspring, depending on $$\beta $$, while equal-fit agents reproduce one offspring. In terms of cultural evolution, agents potentially imitate the social behaviour of successful individuals or role models. Here, as $$\beta $$ approaches 0, agent *i* becomes unable to access information about the payoff of *j*, and thus decides whether to switches to *j*’s strategy by tossing a coin. Note that when $$0< \beta < 2$$, agent *i*’s imitation of *j*’s strategy depends not only on the relative payoff (i.e., fitness) of *j*, but also on $$\beta $$ (i.e., the noise level). Under such levels of noise, if the payoff difference is small (e.g., less than 2), then agent *i* occasionally does not imitate its role model (i.e., does not set $$ CCC _i$$ to be equal to $$ CCC _j$$). Another reason for *i* not to imitate its role model is mutation. More specifically, after each generation, a small fraction of conditional cooperators undergo mutations in the reproduction process, meaning that they miscopy the conditional cooperative strategy of their role models^67^. For more details on mutation parameters, see the Simulations section.

So far, we discussed how conditional cooperators update their social behaviour after each generation. Next, we turn our attention to the altruists in the population. To this end, we introduce a parameter, *w*, specifying the degree of “stubbornness” of altruists, i.e., the probability with which they do not imitate their role models. With this parameter, the case in which the altruists are stubborn (e.g., cultural leaders or freedom fighters) can be modeled by setting $w=1$. On the other hand, the case in which the altruists may imitate their role models can be modeled by setting $w<1$. In the latter case, the altruists undergo a selection process similar to that of conditional cooperators with probability $(1-w)$.

## Simulations

In the simulations, the population size is $$N = 100$$. A certain percentage of the population consists of altruists, while every other agent *i* is a conditional cooperator whose $$ CCC _i$$ value is drawn from $$\{0,\ldots ,N\}$$ uniformly at random. All agents play a repeated PGG with an initial endowment $$e = 10$$ units given to each agent in each round. Every conditional cooperator *i* determines its contribution following Eq. (1), receives its payoff following Eq. (2) and, after each generation, updates it strategy (i.e., $$ CCC _i$$) following Eq. (3) with 5% mutations, implying that 5% of conditional cooperators miscopy their role model’s strategy. These mutations are created by adding to the updated $$ CCC _i$$ a random value drawn from a Gaussian distribution with mean = 0 and s.d. = 5 (max = 50; min = −50). If the mutated $$ CCC _i$$ is greater than *N*, it is rounded off to *N*; if the mutated $$ CCC _i$$ is negative, it is rounded off to 0. Note that if a conditional cooparator *i* happens to have $$CCC_i=0$$ due to mutation, it does not make *i* an altruist; it just means that *i* will cooperate in the next round regardless of how many others do so. However, *i* would still undergo the selection process after the current generation, unlike altruists who would only undergo this process with probability $$(1-w)$$.

Simulations are carried out with the contribution cost $$u = 0.5$$ and the enhancing factor $$h = 1.5$$, while varying the percentage of altruists $$\alpha \in \{10\%,20\%, \ldots , 50\%\}$$, the noise level $$\beta \in \{0, 0.1, \ldots , 1, 2, \infty \}$$, and the probability with which altruists do not imitate their role models, $$w \in \{0.1, 0.2, \ldots , 0.6, 1\}$$. For example, given $$w = 0.6$$, every altruist cooperates in the first generation, and then, after each generation, undergoes the selection process with probability $$(1-0.6)$$. The simulations were carried out for each experimental condition (i.e., fixed set of parameters: *N*, *e*, *u*, *h*, $$\alpha $$, $$\beta $$, *w*) for 20,000 generations, and were repeated 30 times to compute the average behaviour of the population dynamics. We focused on three outcome measures: (i) the evolution of cooperation levels across generations; (ii) the asymptotic behaviour of the evolution of cooperation; (iii) the distribution of the $$ CCC _i$$ values in the 20,000th generation. The cooperation level in each generation was measured as the percentage of agents who contributed to the common pool.

## Results

For all experiments that involve altruists, we set the cooperation level in the first generation to be equal to the percentage of altruists in the population. Moreover, for all results presented in this section, the contribution cost is $u=0.5$, the enhancing factor is $h=1.5$, and the population size is $N=100$. As we will show later on in this section, these parameters allow us to reproduce the broad trend observed in PGG experiments^2^. For details about the Matlab code required to run the simulations and produce all figures, see the Supplementary Material.

**Figure 1:**
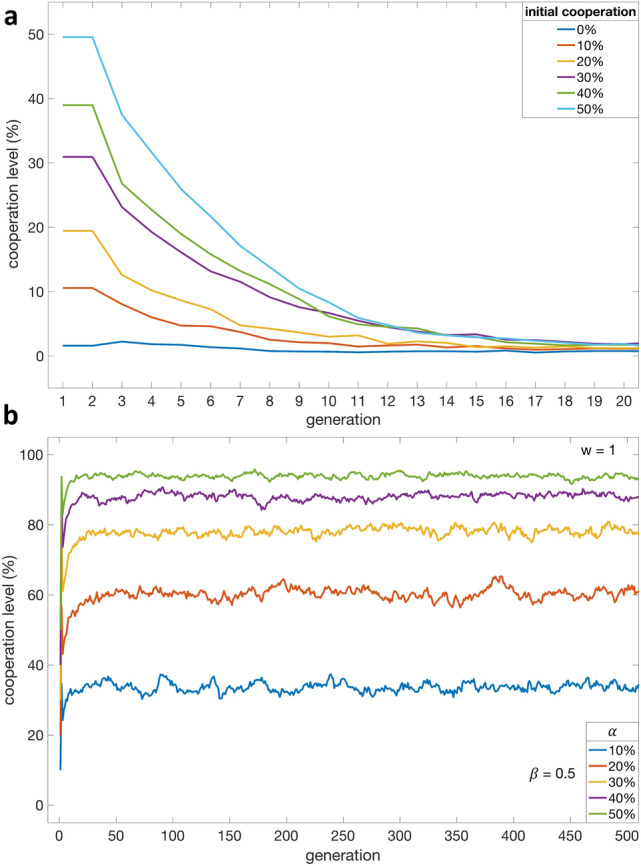
The evolution of conditional cooperation. The x-axis represents the generation, the y-axis represents the cooperation level, and each color-coded trajectory depicts the evolutionary dynamics of cooperation under different experimental conditions, given $\beta = 0.5$. **a**, Evolution of cooperation in the absence of altruists, given different percentages of cooperation decisions made in the first generation. **b**, Evolution of cooperation given different percentages of altruists present across generations when $w=1$.

Let us first analyze what happens in a repeated public goods game when the noise level is $$\beta = 0.5$$ and when every agent *i* is a conditional cooperator whose $$ CCC _i$$ value is drawn from $$\{0,\ldots ,N\}$$ uniformly at random. Starting with different percentages of cooperation decisions in the initial generation, Fig. 1a shows the evolution of cooperation over the first 20 generations. As can be seen, regardless of the initial cooperation level, cooperation declines over 20 generations, resembling behavioural regularities observed in repeated public goods games^2^. On the other hand, the presence of a few free-riders in the initial generation triggers free riding in subsequent generations. This is because agents behave like conditional cooperators and imitate their role models’ strategies after each generation.

Let us now consider what happens under the same experimental conditions, but when altruists are introduced into the population and continue to cooperate unconditionally across generations; this experimental condition can be obtained by setting $w=1$. We will analyze the evolution of cooperation given different percentages of altruists, $\alpha$. Since the population dynamics stabilize after a few hundred generations, we will focus on the evolution of cooperation during the first 500 generations; later on, we will analyze asymptotes drawn for the last 5,000 generations out of 20,000. As can be seen in Fig. 1b, for every $\alpha \in \{10\%, 20\%, 30\%, 40\%, 50\%\}$, the population establishes a cooperation level greater than $\alpha$ within a few generations. While this in itself is not surprising, the degree to which cooperation increases is indeed noteworthy, especially when it reaches a level $> 2\alpha$. For instance, starting with $\alpha = 20\%$, the population eventually reaches a cooperation level of about $65\%$. Similarly, starting with $\alpha = 30\%$, the cooperation level eventually reaches $80\%$. This is because the altruists are able to create a critical level of cooperation that exceeds the threshold of most conditional cooperators, while the presence of moderate levels of noise (i.e., $\beta = 0.5$) slows down the proliferation of free-riders.

**Figure 2:**
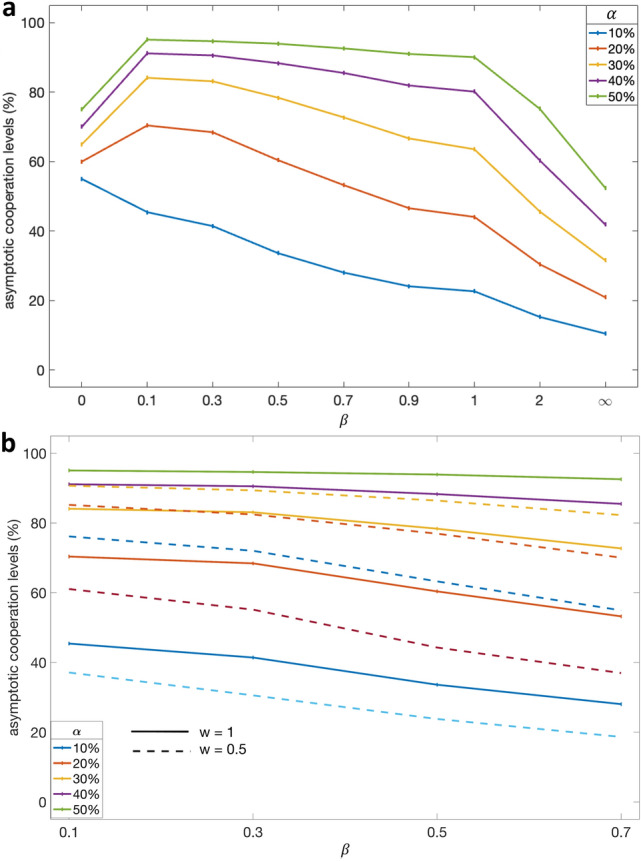
Asymptotic cooperation levels. **a**, The x-axis represents the percentage of altruists, the y-axis represents the average cooperation level over the last 5000 generations out of 20,000 with $w=1$, with each color corresponding to a different level of noise, $\beta$. **b**, The same as (**a**) but for $w=0.5$ instead of $w=1$. **c**, The same as (**a**) but while varying *w* (x-axis) and fixing $\alpha =30\%$.

Next, we analyze how the evolution of cooperation is affected by the noise level $\beta$. Recall that a smaller value of $\beta$ corresponds to a greater level of noise. As such, when $\beta \rightarrow \infty$, noise is minimized, implying that agent *i* always imitates its role model’s strategy, and always determines whether to cooperate based solely on $CCC_i$ and $N_C$. On the other hand, when $\beta \rightarrow 0$, noise is maximized, implying that the agents’ decisions, be it about cooperation or about imitation, are determined entirely based on a coin toss. By varying the noise level between these two extremes, we control the degree to which the agent’s decisions are based on random chance; see Eq. (1) and Eq. (3) for more details. With this in mind, given different percentages of altruists and different levels of noise, Fig. 2a shows the asymptotic cooperation levels in the last 5,000 generations out of a total of 20,000 (error bars were omitted since they were extremely small). As shown in this figure, decreasing the noise level (i.e., increasing $\beta$) results in lower levels of cooperation, regardless of the percentage of altruists. The only exception is when noise is maximized (i.e., when $\beta = 0$), in which case the cooperation level equals $\alpha +(N-\alpha )/2$. This is because, in the absence of information about social interactions, the altruists always cooperate, while other agents contribute to the common pool by tossing a coin. On the other extreme, when noise is minimized (i.e., when $\beta = \infty$), the cooperation level equals the percentage of altruists. In other words, apart from the altruists, all agents end up free riding, since they have perfect information about the payoff advantage of their role models who are mostly free-riders.

Next, we analyze what happens when we relax the assumption that the altruists are stubborn, i.e., when we set $w<1$ instead of $w=1$. As shown in Fig. 2b, cooperation levels drop slightly when setting $w=0.5$ instead of $w=1$, e.g., given $\alpha =30\%$ and $\beta =0.3$, cooperation levels drop from 86 to 77%. This drop is due to the fact that altruists are no longer stubborn, but rather change their social behaviour with certain probability after each generation. As a result, the number of conditional cooperators increases, and these agents often imitate their role model’s behaviour, leading to the observed drop in cooperation levels. Nevertheless, these results demonstrate that cooperation can still be established even when $w=0.5$, i.e., even if every altruist undergoes the selection process with 50% probability. This crucial result indicates that the altruists need not be stubborn across generations for cooperation to be established in a population of conditional cooperators.

**Figure 3:**
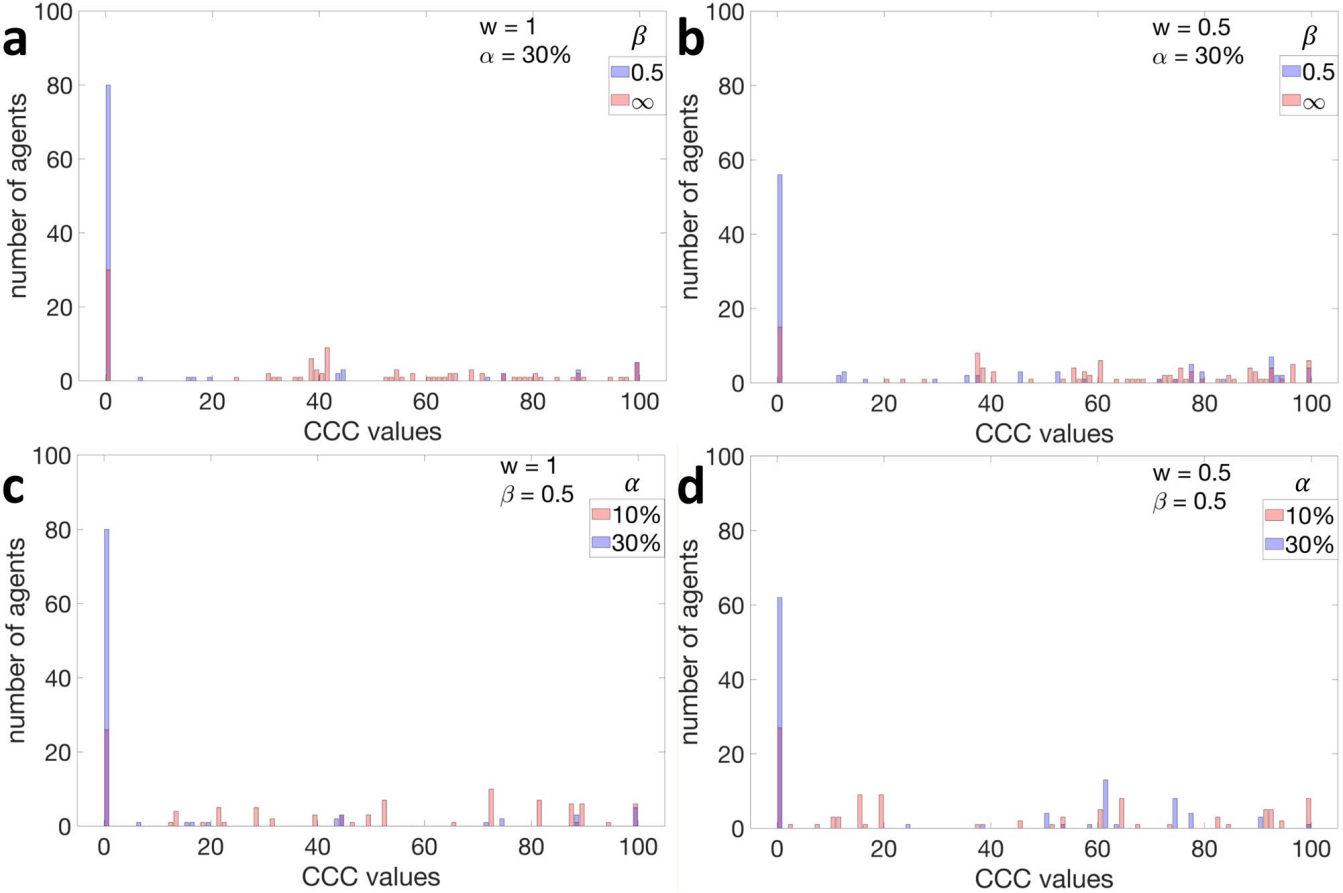
Distribution of the conditional cooperative criterion (CCC) values. Every altruists is born into the initial population with a conditional cooperative criterion $$ CCC _i = 0$$, while every conditional cooperator *k* is born with $$ CCC _i \sim \{0,\ldots ,N\}$$. The figure depicts the distribution of all CCC values in the 20,000th generation for different percentages of altruists ($$\alpha $$), different levels of noise ($$\beta $$), and different values of *w*. **a**, Results when $$\alpha = 30\%$$, $$\beta \in \{0.5,\infty \}$$, and $$w=1$$. **b**, The same as (**a**) but for $$w=0.5$$ instead of $$w=1$$. **c** Results when $$\alpha \in \{10\%,30\%\}$$, $$\beta = 0.5$$, and $$w=1$$. **d**, The same as (**c**) but for $$w=0.5$$ instead of $$w=1$$.

Having discussed the cooperation levels, let us now discuss the distribution of the conditional cooperative strategies, i.e., the CCC values. Starting with a population in which every conditional cooperator *i* is born with $CCC _i \sim \{0,\ldots ,N\}$, Fig. 3a compares the distribution of the CCC values in the 20,000th generation in two different situations. The first is where $\alpha = 30\%$, $\beta = 0.5$ and $w=1$ (a situation that yields high cooperation levels, as we have seen in Fig. 2a), and the second is where $\alpha = 30\%$, $\beta = \infty$ and $w=1$ (a situation that yields low cooperation levels). In the first situation, nearly 80% of agents adopt a CCC value equal to 0, while in the second situation, nearly 25% do so, with the majority of the population adopting CCC values greater than 50. Figure 3b shows what happens when $w=0.5$ instead of $w=1$. In this case, the broad trend remains unchanged; when $\beta = 0.5$ most CCC values equal zero, and when $\beta = \infty$ most CCC values are greater than 50. Finally, given a noise level $\beta = 0.5$, Fig. 3c and d show how the distribution of CCC values is affected by decreasing the percentage of altruists from $30\%$ to $10\%$. As shown in these figures, regardless of the value of *w*, the percentage of agent who adopt a CCC value equal to 0 drops significantly when $\alpha$ changes from $30\%$ to $10\%$. This demonstrates that a critical amount of altruism is needed to influence the majority of conditional cooperators.

## Discussion

In this study, we set out to answer a fundamental question: Can cooperation be established without external enforcement when individual and collective interests are in conflict? If so, under what conditions? We showed that cooperation is not established either when the population consists of conditional cooperators only Fig. 1a or when it consists of both conditional cooperators and altruists but in the absence of noise (Fig. 2a). This shows that, perhaps counterintuitively, injecting altruists into a population of conditional cooperators is not sufficient to establish stable cooperation. On the other hand, in the presence of moderate noise levels, the combination of a few altruists and conditional cooperators can establish and sustain high levels of cooperation Fig. 1b, and increasing the number of altruists would result in an increase in cooperation levels (Fig. 2a). Crucially, this finding holds even when some of the altruists change their social behaviour across generations (Fig. 2b). Finally, we examined the extreme case where noise is maximized, showing that cooperation dynamics in this case are entirely driven by both noise and altruists (Fig. 2a).

Let us now discuss why, in the presence of a few altruists and a moderate level of noise, cooperation can be established and sustained. To start with, the altruists’ actions trigger cooperation by the conditional cooperators. Before discussing how this cooperation is sustained, recall that each agent in our model is randomly paired with another agent, after which it updates its strategy following Eq. (3). In the presence of altruists, agents are less likely to be paired with role models, i.e., with agents who free ride. Moreover, in the presence of noise, agents are less likely to imitate the role models’ strategies, since they have imperfect information about the payoff of their role model, and are thus less likely to recognize the advantage of copying their strategy. Consequently, as we have shown in Fig. 3, the population becomes increasingly dominated by agents with low CCC values which, in turn, makes the agents more likely to imitate positive reciprocators. Under these conditions, the agents’ social learning is influenced not only by the role models’ payoffs, but also by the frequency of the behaviour observed in the population, suggesting that both payoff-biased transmission and conformist-biased transmission are influencing social learning. As a result, the proliferation of free-riders slows down, thereby sustaining the cooperation that was triggered by the altruists. Finally, let us explain why cooperation is not established in the absence of noise. This is simply because the agents in this case would always imitate their role models’ social behaviour, leading to the proliferation of free riding. Having said that, it should be noted that our mechanism required a considerable number of altruists for it to work. For example, under certain levels of noise, it may require 20% or even 30% of the population to be altruists.

Next, we discuss what happens when a free-riding mutant (i.e., an agent *i* such that $$ CCC _i = N$$) is born into a population where altruists are present and noise levels are moderate. In this case, agent *i* does not proliferate in the population due to the above reasons, i.e., because individuals rarely match with *i*, and when they do, they are less likely to imitate *i*. Note that, when noise levels are low, individuals are likely to recognize the superior fitness of *i* and hence imitate *i*, ultimately leading to the proliferation of free-riders. On the other hand, when noise levels are high, individuals’ cooperation decisions and imitations become essentially random, thereby limiting the influence that *i* has on the stability of cooperation.

Our proposed mechanism is novel compared to the punishment-based alternative. More specifically, the core idea behind punishment is to reduce the free-riders’ payoffs, thereby preventing them from reproducing at a faster rate than the cooperators. However, such mechanisms suffer from a number of limitations. First, they reduce the overall social good^2,3,6^. Second, they are sensitive to errors that involve either punishing cooperators or not punishing free-riders^23,30^. Third, they are ineffective when counter-punishments are possible, i.e., when the free-riders are able to retaliate against the punishers^3^. Fourth, their effectiveness is reduced when the population is dominated by either free-riders or second-order free-riders^41^. Fifth, pool punishment relies on perfect monitoring, which is impractical or infeasible in many cases^21^. Instead of punishing free-riders, an alternative mechanism is to reward cooperators^53^. However, it has been argued that rewards are insufficient to sustain cooperation^83^.

Our model has several merits. First, it avoids the limitations of punishment-based mechanism. Second, it avoids the common assumption that agents are rational and selfish^84^. Third, it allows the population to be influenced by conformist-biased transmission—a form of social learning often observed in human societies^85^. Fourth, our model relies on three assumptions that often hold in the real world: (i) the majority of the population are conditional cooperators, as commonly observed in the field^17,76^; (ii) cooperative dynamics are stabilized by social noise as observed elsewhere^86,87^; (iii) a few individuals (the altruists) can influence the majority (the conditional cooperators), as is the case with cultural leaders who trigger tipping-point phenomena observed in social dynamics^77–80^. Despite its advantages, the proposed mechanism may not work in small groups, where individuals are more likely to access perfect information about the payoffs of their role models, and are thus more likely to imitate the social behaviour of negative reciprocators or free-riders, thereby leading to free riding. Finally, although the model helps us understand the conditions under which cooperation can be established without external enforcement, it does not specify the conditions under which conditional cooperation evolves endogenously; this latter point is certainly worth exploring in future research. Another promising future direction is to explore the case where individuals are embedded in a spatial network, which is often the case in the real world.

In summary, we demonstrated that a population of conditional cooperators (without altruists) does not establish cooperation (Fig. 1a). Furthermore, we demonstrated that, even with the injection of altruists, stable cooperation is still not always established—a counterintuitive finding, especially since one would imagine that conditional cooperators would (by definition) cooperate in the presence of altruists (see Fig 2a when noise level is low). Finally, we identified a condition under which altruists can trigger and sustain cooperation; this condition is the presence of moderate noise levels in social interactions, which are commonly observed in the real world ^82^. Our findings suggest that, in order to facilitate self-governance, societies should focus on creating a critical amount of trust to harness the conditional nature of its members. More broadly, we show that it is indeed possible to establish cooperation without punishment.

## Supplementary Information

Supplementary Information 1.

Supplementary Information 2.

## Data Availability

All data, as well as the Matlab code required to run the simulations and produce the plots, are provided in http://doi.org/10.5281/zenodo.7442767this link.

## Code availability

The code used to run the simulations and produce the figures is freely available in the Supplementary Material.
