# Supplementary material for: Cooperation without punishment
Source: Sci Rep. 2023 Jan 21;13:1213. doi: 10.1038/s41598-023-28372-y (PMC9867775; doi:10.1038/s41598-023-28372-y)
Supplement: Supplementary file 1 — Supplementary Information 1. [file Third party punishment (TPP) cover letter.pdf › pgg_alt1/Third party punishment (TPP) cover letter.pdf]

جامعة نيويورك أبوظبي
NYU | ABU DHABI

September 20, 2020

Dear Editors,

It is our pleasure to submit our paper, "*Norms of fairness influence trust in human-machine interaction*", for your kind consideration in Nature.

Ample work has documented that the functioning of human societies is undergirded by norms of prosocial behavior, a prerequisite of which is trust in anonymous strangers. We ask if we already have the foundations for similar norms to trust AI, or if we shall adapt to achieve them? To answer this fundamental question, we ground our work in a long tradition studying the underpinnings of trust and cooperation among people, with particular attention to the role of third-party punishment; see, e.g.:

- Fehr, E. & Gachter, S. "*Altruistic punishment in humans.*" **Nature** (2002).
- Nowak, M. "*Five rules for the evolution of cooperation.*" **Science** (2006).
- Rand, D., et al., "*Positive interactions promote public cooperation.*" **Science** (2009).
- Jordan, J. "*Third-party punishment as a costly signal of trustworthiness.*" **Nature** (2016).

We bring this literature together with a recent, interdisciplinary direction studying human-machine interaction:

- Crandall, J.et al. "*Cooperating with machines.*" **Nature Communications** (2018).
- Rahwan, I. et al. "*Machine behaviour.*" **Nature** (2019).
- Ishowo-Oloko, F., et al. "*Behavioural evidence for a transparency—efficiency tradeoff in human–machine cooperation.*" **Nature Machine Intelligence** (2019).

In three behavioral experiments, we provide evidence for the following claims: (1) when bots are fair or punish the unfair, they inspire trust, albeit to a lesser extent compared to people; (2) when people are fair to bots or punish those who are unfair to bots they signal trustworthiness, but to a lesser extent compared to when people are the beneficiaries; (3) people's belief about the consensus held regarding norms of fairness and third-party punishment influences the trust that can be gained from these behaviors, regardless of whether the interaction partners are bots or people; finally (4) manipulating peoples' belief about norm-consensus can be an effective tool to increase the trust-gain of bots who are fair. These findings not only furnish practical guidance for enhancing human-machine interaction, but also provide the first experimental evidence linking norm consensus to the costly signaling of private information.

We are grateful for your kind consideration, and would like to propose the following potential reviewers:

- Michael Rovatsos <mrovatso@inf.ed.ac.uk>, University of Edinburgh
- Minjea Kim <minjaekim@rice.edu>, Rice University
- Robb Willer <willer@stanford.edu>, Stanford University
- Jillian Jordan <jillianjordan@hbs.edu >, Harvard Business School
- Donghyun Choi <dannychoi@pitt.edu>, University of Pittsburgh
- Joachim Krueger <joachim_krueger@brown.edu>, Brown University

Sincerely,

Kinga, Ana, Wendi, and Talal
